# Diamond Relaxometry as a Tool to Investigate the Free Radical Dialogue between Macrophages and Bacteria

**DOI:** 10.1021/acsnano.2c08190

**Published:** 2023-01-11

**Authors:** Kaiqi Wu, Linyan Nie, Anggrek C. Nusantara, Willem Woudstra, Thea Vedelaar, Alina Sigaeva, Romana Schirhagl

**Affiliations:** † Department of Biomedical Engineering, University of Groningen, University Medical Center Groningen, Antonius Deusinglaan 1, 9713 AV Groningen, The Netherlands

**Keywords:** fluorescent nanodiamonds, free radicals, *S. aureus*, bacterial infections, diamond magnetometry, microorganism antioxidative defense

## Abstract

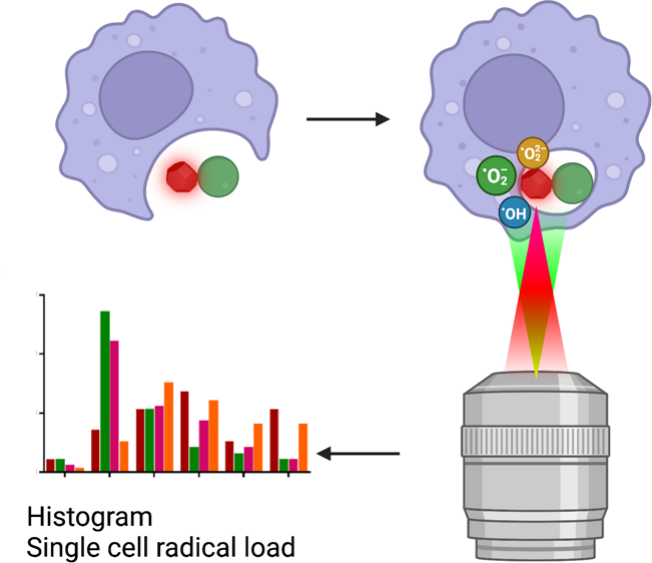

Although free radicals, which are generated by macrophages play a key role in antimicrobial activities, macrophages sometimes fail to kill *Staphylococcus aureus* (*S. aureus*) as bacteria have evolved mechanisms to withstand oxidative stress. In the past decades, several ROS-related staphylococcal proteins and enzymes were characterized to explain the microorganism’s antioxidative defense system. Yet, time-resolved and site-specific free radical/ROS detection in bacterial infection were full of challenges. In this work, we utilize diamond-based quantum sensing for studying alterations of the free radical response near *S. aureus* in macrophages. To achieve this goal we used *S. aureus*-fluorescent nanodiamond conjugates and measured the spin–lattice relaxation (T1) of NV defects embedded in nanodiamonds. We observed an increase of intracellular free radical generation when macrophages were challenged with *S. aureus*. However, under a high intracellular oxidative stress environment elicited by lipopolysaccharides, a lower radical load was recorded on the bacteria surfaces. Moreover, by performing T1 measurements on the same particles at different times postinfection, we found that radicals were dominantly scavenged by *S. aureus* from 80 min postinfection under a high intracellular oxidative stress environment.

## 1 Introduction

Free radicals play a key role in many biological processes including cell communication, aging, and cell maturation as well as immune responses. They are also crucial for many pathological conditions including cancer, cardiovascular diseases, and bacterial and viral infections. However, due to the odd number of electrons in free radicals, they are extremely unstable and highly reactive. Since free radicals are present in low concentrations in biological samples, a highly sensitive method is desired to detect them.^1^ To our knowledge, there is currently no technique to specifically measure the nanoscale distribution of free radicals in real time. Traditional methods are usually based on fluorescent dyes, which can measure total ROS levels. Nevertheless, these dyes bleach over time and are not suitable for long-term measurements. Additionally, they reveal the history of the sample rather than the current status. Studies reported reactive oxygen species (ROS) measurement within 1–3 h after treatment.^2,3^

In macrophages, radicals are involved in killing pathogens such as bacteria. Macrophages recognize and engulf bacteria into phagosomes. This process eliminates intracellular pathogens and helps maintaining cellular homeostasis. Functional phagocytosis is crucial for immune responses by killing many life-threatening bacteria in the human body. Phagocytosis also plays an important role in developing antimicrobial therapies and combating fast developing antibiotic resistant bacteria.^4,5^ There are several reasons to investigate this field such as rare but devastating diseases related to defects in phagocytosis or killing of bacteria. Further some of the most pathogenic bacteria developed strategies to escape killing during phagocytosis.^4,6,7^

After the uptake of bacteria, the phagosome matures and the environment becomes acidic (initially around pH 6 and further decreasing to around 4.5 in phagolysosomes). This process is closely related to the complex killing of bacteria inside macrophages. Apart from phagocytosis, there are many other factors that could lead to bacteria killing in immune cells.^4,7^ For instance, antibiotic peptides, hydrolytic enzymes, or proinflammatory factors can contribute.^8^ Among these factors, a low pH and the generation of free radicals are considered the most important elements.^9^ Killing bacteria by free radicals is caused by superoxide (O_2_^–^*), which is produced by NADPH oxidase (NOX) early in the phagocytic process.^10,11^ However, recent studies reported that several pathogens can escape from phagosomes and thus establish residence in the cytoplasm of host cells.^12^ Especially for *Staphylococcus aureus* (*S. aureus*), lower pH plays a key role for them to survive.^7,13,14^ In the early phase of phagosome maturation, in the low pH (pH 5.0–6.0) environment inside early phagolysosomes, most enzymes (like cathepsins, proteases, lysozymes) are active. During this process, NADPH oxidase generates O_2_^–^*. At a much lower pH of 4.5 in phagolysosome, these enzymes aggregate and are inactivated.^15,16^ Another reason that *S. aureus* can escape from phagocytosis is that *S. aureus* has a golden pigment called staphyloxanthin. This pigment works as an antioxidant, which can decrease the free radical load.^17^ Insufficient concentrations of free radicals in macrophages not only fail to kill bacteria but also induce higher stress, leading to host cell damage. Under this circumstance, a low number of free radicals can cause more harm for the host cells.^18^

The complete process of how pathogens escape from phagosomes and survive in macrophages remains unclear. One of the unknown factors is the exact microenvironment that bacteria are exposed to. To understand the role of free radicals in *S. aureus* escaping from macrophages, we used fluorescent nanodiamonds (FNDs) as a probe to map free radicals. To this end, we use diamonds containing defects called nitrogen vacancy (NV) centers. These defects change their optical properties based on their magnetic surrounding. Since it is easier to measure optical signals than small magnetic signals themselves, this technique is extremely sensitive.^19^ NV centers have been utilized in physics for many different applications including of measurements of single electrons^20^ or nuclear spins,^21,22^ magnetic characterization of materials under high hydrostatic pressures,^23−25^ measurements of 2-dimensional materials,^26^ magnetic domain walls,^27^ magnetic fields of nanoparticles,^28^ or the presence of molecules on the diamond surface.^29,30^ However, NV centers are not used routinely in biological applications. In this work, a specific mode of diamond magnetometry called relaxometry was used.^31^ This technique has been used to provide a nanoscale-resolution measurement for free radicals in yeast cells, dendritic cells, epithelial cells, kidney cells, sperm cells as well as macrophages.^32−37^ With NV centers inside FNDs, they can “feel” magnetic noise and convert it into an optical signal. Thus, we can detect the spin noise from free radicals using a home-built magnetometer.

**Figure 1:**
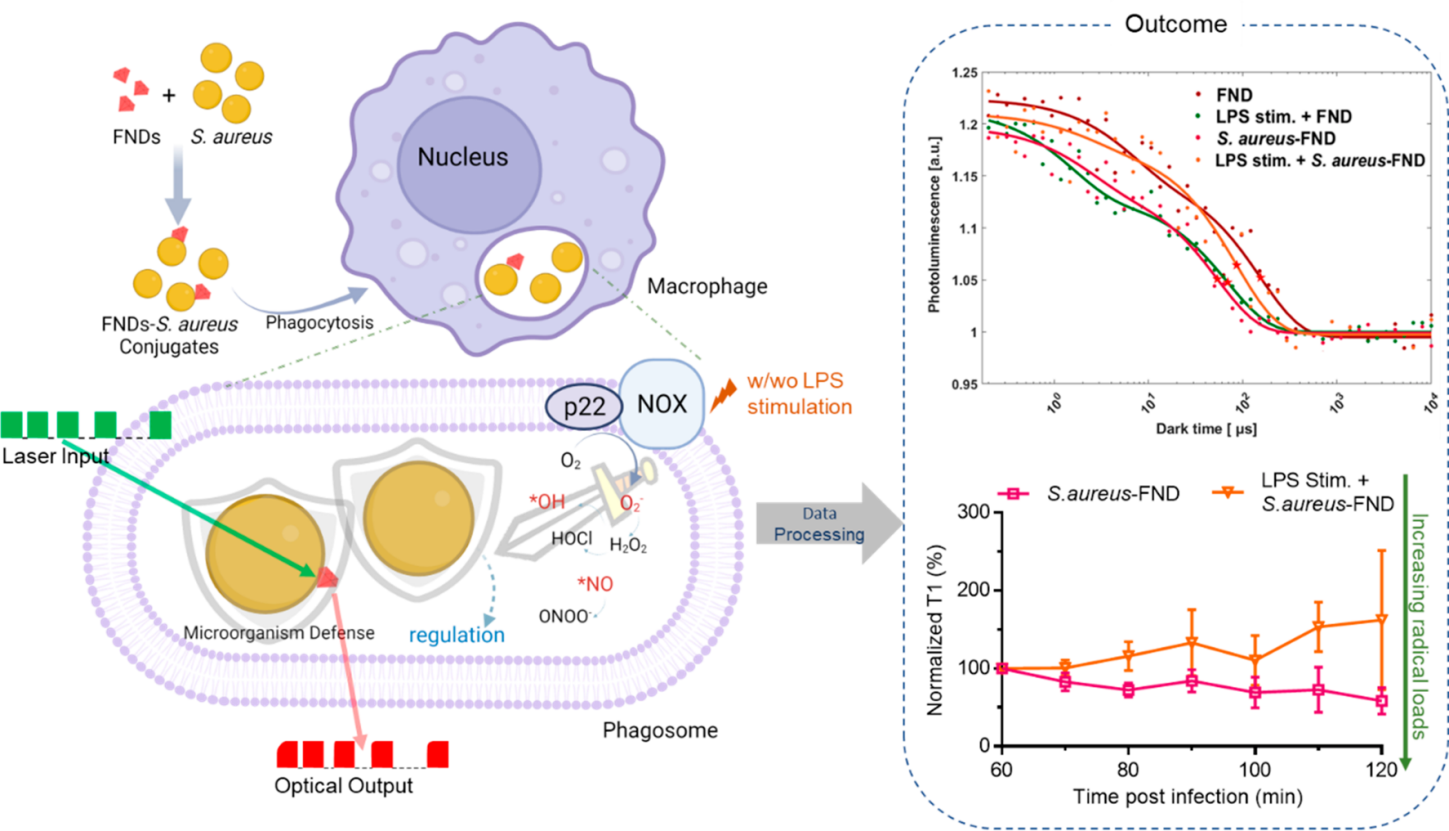
Schematic representation of applying relaxometry to probe free radicals of J774 macrophages upon *S. aureus* infections using bacteria–FND conjugations.

In this paper, FNDs were conjugated to *S. aureus* by physical adsorption. This way we can measure locally on the surface of bacteria while they are phagocytosed (Figure 1). Moreover, how and when bacteria modulate the high intracellular oxidative stress was elucidated dynamically.

## 2 Results and Discussion

### 2.1 Characterization of *S. aureus*–Green Fluorescent Protein (GFP)–FNDs Conjugates

**Figure 2:**
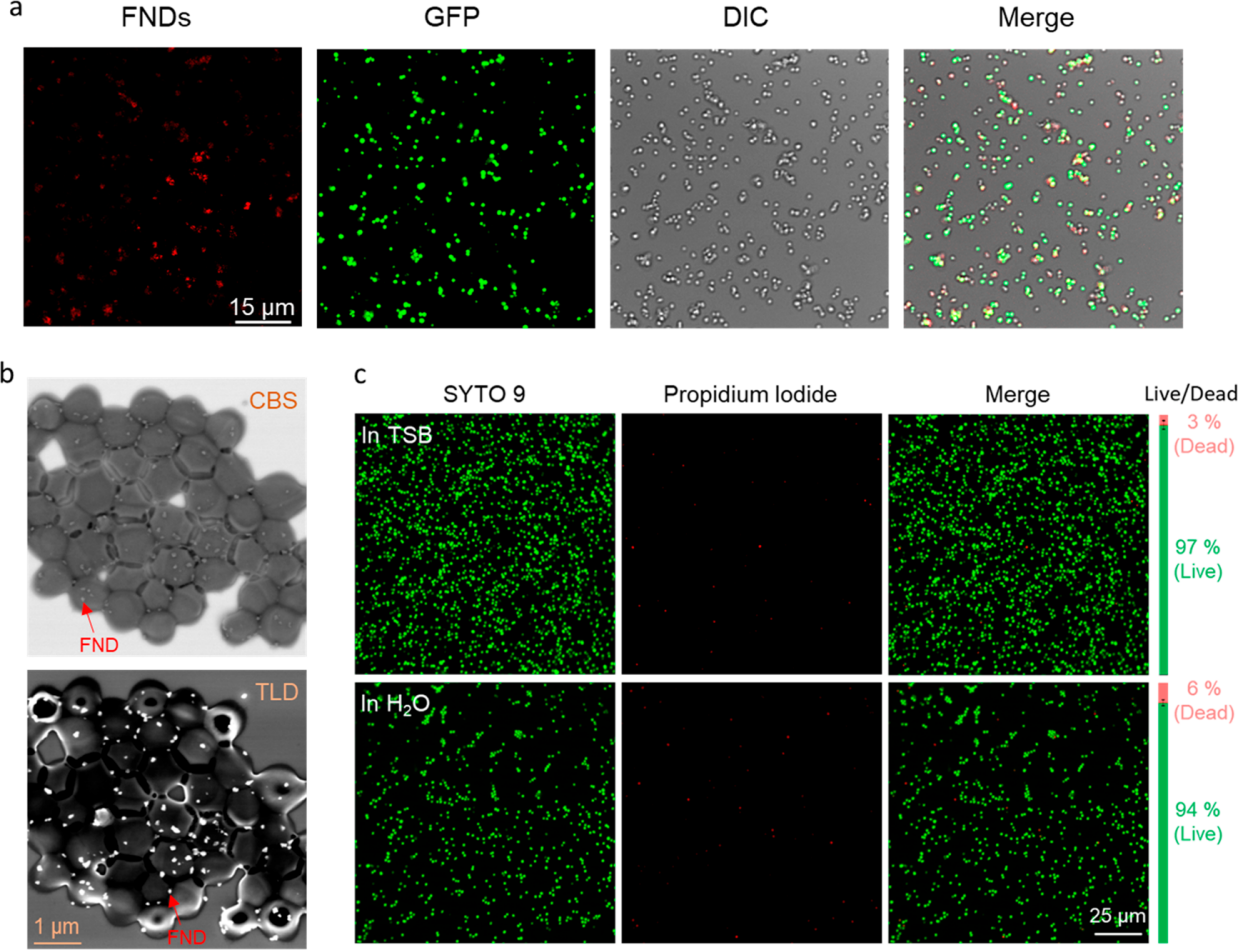
Characterization of *S. aureus*–FNDs conjugates. (a) Confocal images of *S. aureus*–GFP–FNDs conjugates. Red, FNDs; Green, GFP signals from *S. aureus–GFP*; DIC, differential interference contrast microscopy. (b) SEM images of *S. aureus*–FNDs conjugates. The upper image was recorded by CBS, while TLD was used to achieve the high-contrast images (the lower picture). The brighter dots are FNDs, and one of them is marked with red arrow as an example, and the darker grapes are *S. aureus*. (c) Viability test of *S. aureus* under FND conjugation conditions (*S. aureus* was kept for 2 h in demi water). Fluorescent images of Live/Dead staining assay of *S. aureus* in TSB and demi water, respectively. The green and red bars beside the fluorescent images represent the live and dead ratio for each group. The live/dead ratio was calculated from around 3000 bacteria. The experiment was repeated three times.

Size distribution of the conjugates were measured by DLS (see Figure S1), the average hydrodynamic diameter of FNDs is increased compared to bare FNDs (85–100 nm in demi water, 200–300 nm in 30% TSB, and 400–600 nm in 100% TSB). The dynamic light scattering (DLS) result is consistent with previous results that showed that FNDs will aggregate in a buffer.^38,39^ To avoid aggregation, the *S. aureus*–GFP–FND conjugates were prepared in demi water. The interaction of FNDs with Gram-positive bacterial strains has been studied in the past.^40,41^ These studies denoted that commercial FNDs can conjugate with *S. aureus* without further modification. As shown in the confocal images (Figure 2a), all the FNDs (red signal) colocalized very well with *S. aureus*–GFP (green signal), and more than 50% of *S. aureus*–GFP have FNDs attached to the cell wall (In the representative images, a total of 290/421 *S. aureus*–GFP have red FNDs attached). The electronic microscope images (Figure 2b) also show the FNDs (the gray dots in the CBS-detector image, and the white dots in TLD detector image, marked one FND as an example with a red arrow) that have conjugated with the *S. aureus*. We did not observe any gray dots (FNDs) on the surfaces of *S. aureus* without FND conjugation (Figure S4). The high resolution, full-sized images and imaging details of scanning electron microscopy (SEM) images of *S. aureus*–FND and *S. aureus* are shown in the Supporting Information (Figures S3 and S4). All confocal laser scanning microscopy (CLSM) and SEM images show that there were few free FND particles in the conjugation system, which means that the purification with centrifuging and washing process can help to avoid making T1 measurements on free FND particles.

A Live/Dead staining assay was performed to assess the viability of *S. aureus* under the conjugation condition after 2 h of incubation (Figure 2c). The Live/Dead ratio of *S. aureus* kept in demi water is similar to that of the bacteria in TSB after 2 h, which indicates that *S. aureus* can survive when kept under conjugation condition.

### 2.2 Subcellular location of bacteria-FND conjugates

**Figure 3:**
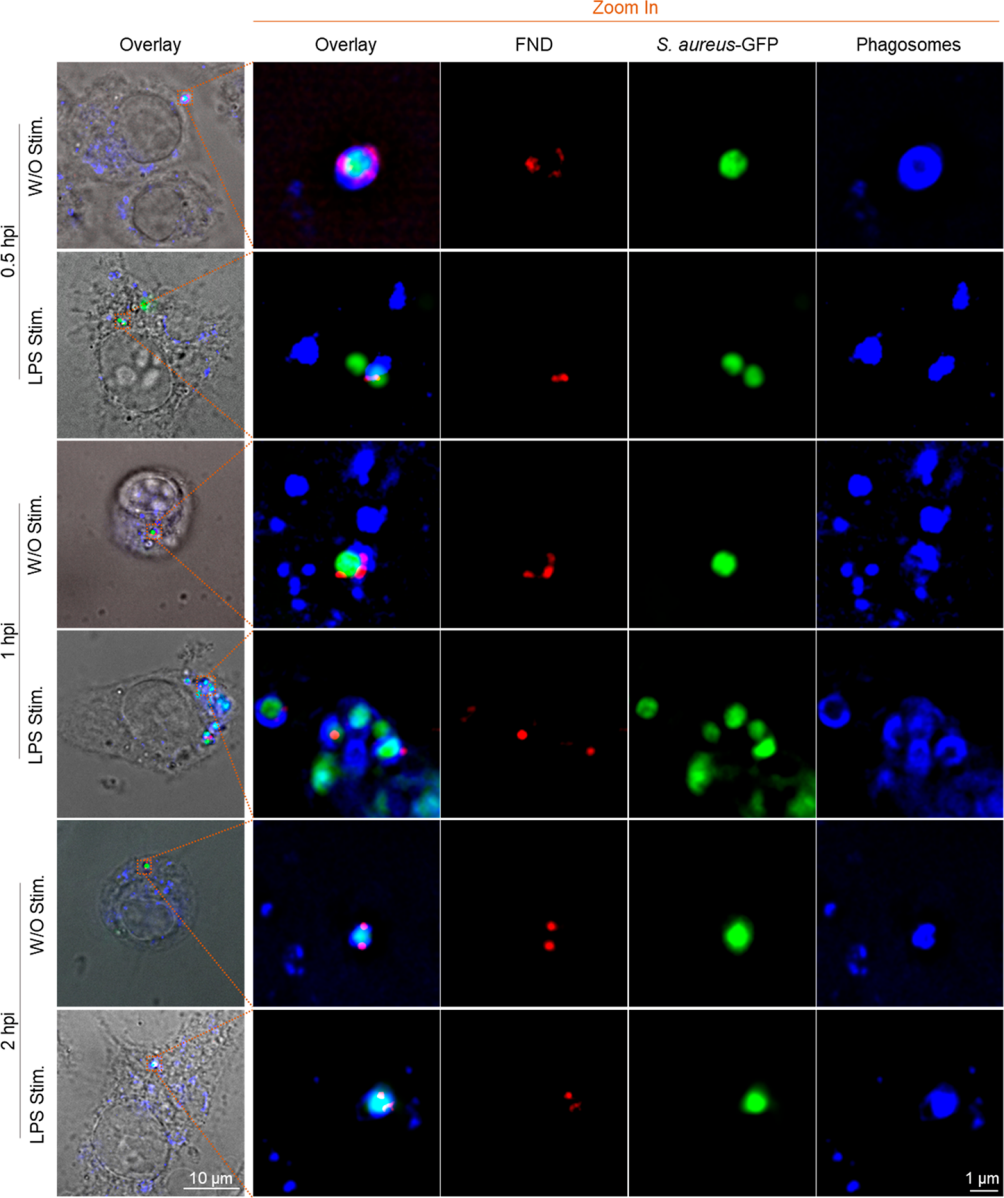
Intracellular location of *S. aureus*–FND conjugates inside macrophages. Macrophages with/without LPS stimulation were first incubated with *S. aureus*–FND conjugates (MOI = 20) for 30 min. After that, the cell medium was removed and replaced with live cell imaging buffer and stained with lysosome tracker (Lysoview 405). Then, cells were imaged directly by confocal after incubating for another 0.5 and 1.5 h.

*S. aureus*–FND conjugates (MOI = 20) were formed to investigate the intracellular free radical response near bacteria. To clarify the subcellular location of *S. aureus*–FND conjugates (where we performed T1 measurements), macrophages were incubated with *S. aureus*–GFP–FND for different times, and Lysoview 405 (in blue) was used to label phago-lysosomes. As shown in Figure 3, after 0.5, 1, and 2 h of incubation, *S. aureus*–GFP–FND conjugates are colocalized with early phagosomes as indicated. To conclude, we can measure the free radical response of macrophages from the surface of phagocytized bacteria, using *S. aureus*–FND conjugates.

**Figure 4:**
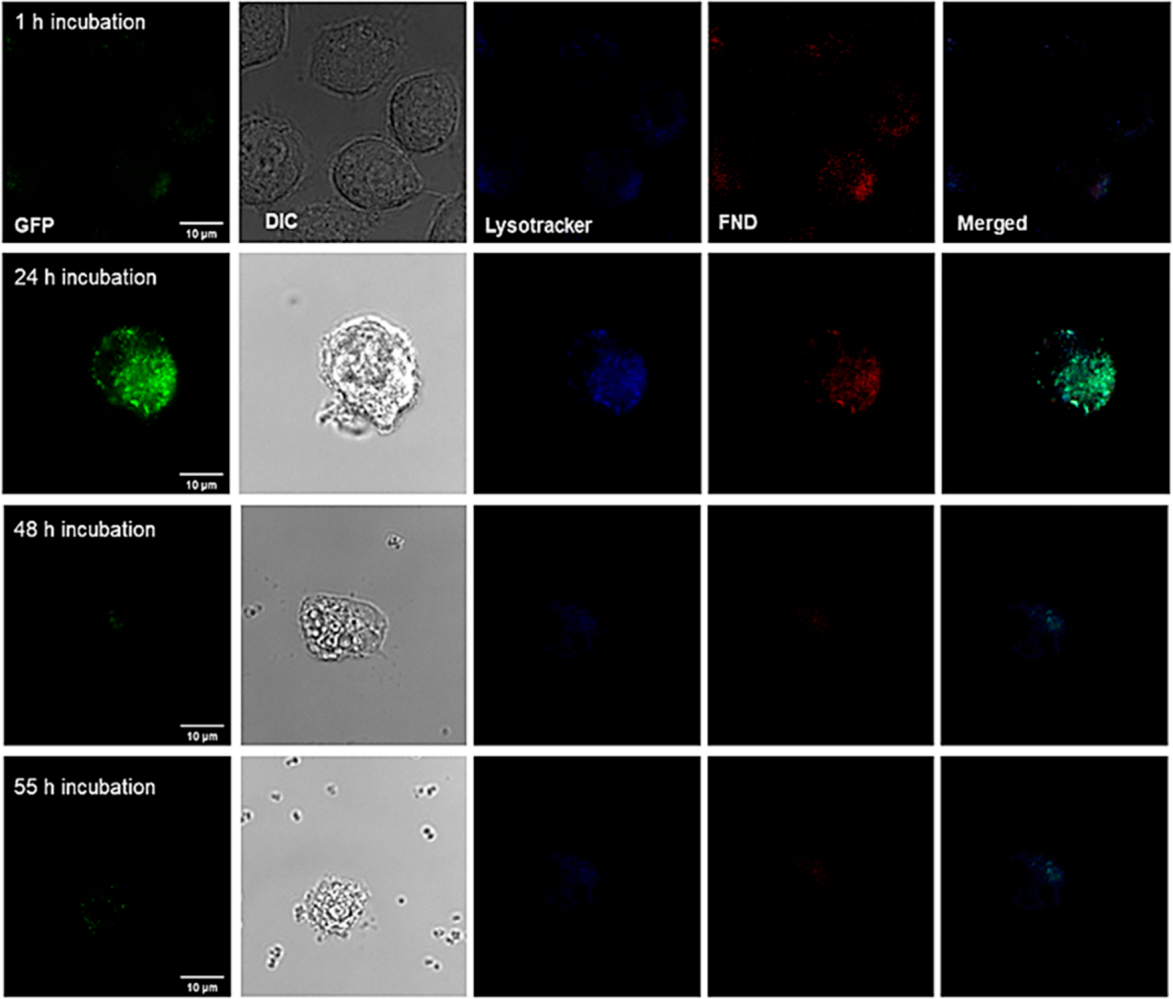
Intracellular location of *S. aureus*–GFP–FNDs conjugates inside macrophages. Macrophages were first incubated with *S. aureus*–GFP–FNDs conjugates (MOI = 20) for 30 min. After that, the cell medium was removed and replaced with live cell imaging buffer and stained with lysosome tracker (Lysoview 405). Then, cells were imaged directly by confocal, after 1, 24, 48, or 55 h of incubation. Green, GFP; blue, Lysoview 405; red, FNDs. Gray: DIC (differential interference contrast). Arrows indicate the locations of *S. aureus*–GFP–FND conjugates.

In addition, *S. aureus* escape from the early phagosomes decides their fate in macrophages.^42^ Once *S. aureus* escaped from early phagosomes, they duplicated inside macrophages and caused macrophage cell death.^43^ At a longer incubation period of 24 h, *S. aureus–GFP–FND* conjugates are found in the phagolysosomes in dead macrophage cells (Figure 4, DIC image). When we increased the incubation time, for example, 48 or 55 h, there were very few *S. aureus*–GFP–FNDs conjugates inside cells, but they are still colocated with phago-lysosomes. Additionally, *S. aureus–GFP–FND* are found released into the environment.

### 2.3 Cellular ROS Measurements

**Figure 5:**
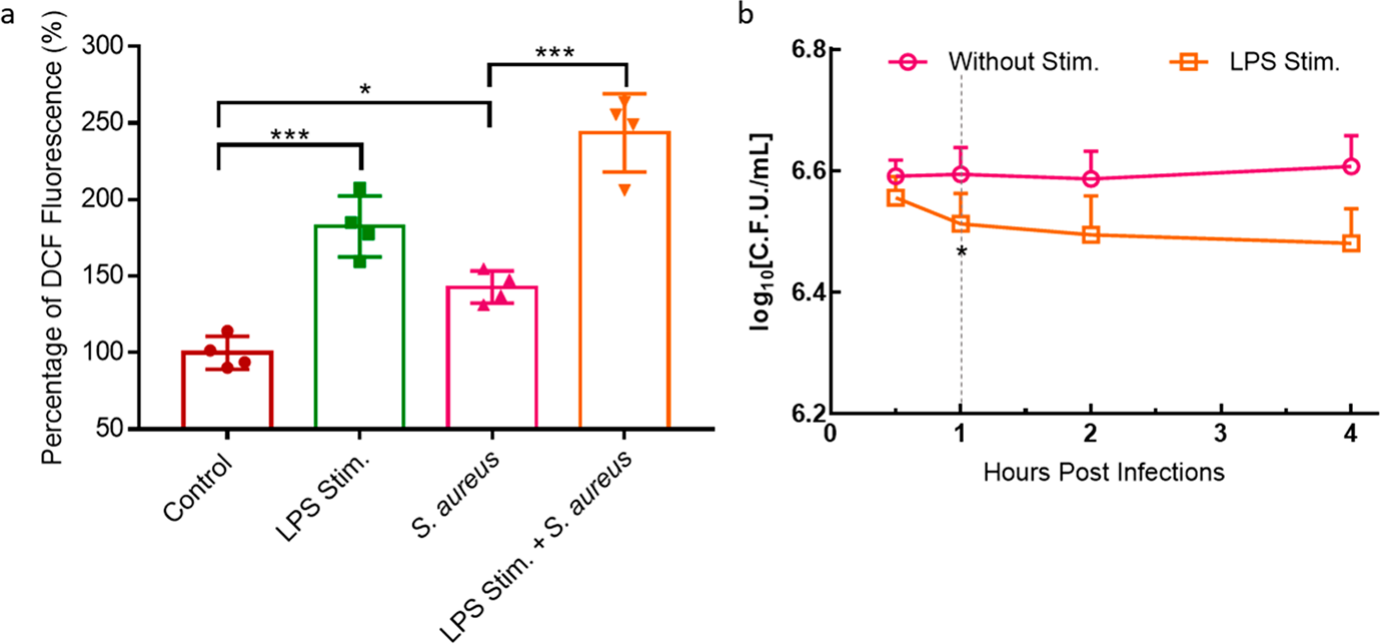
(a) Cellular ROS measurement in macrophages by the DCFDA assay. Macrophages were first stimulated by pretreating with LPS (2 μg/mL) for 24 h. Hereafter FNDs, LPS, *S. aureus*, or *S. aureus*–GFP–FNDs were added and ROS was measured. (b) Survival of *S. aureus* cells after internalization by unstimulated J774 macrophages or macrophages that were stimulated overnight with LPS. The experiments were repeated independently for three to four times with freshly prepared *S. aureus*–FNDs conjugates (MOI = 20). The data was presented as mean ± standard deviation; different symbols stand for individual values. The data were analyzed using a one-way ANOVA among groups; data between each group were analyzed by an unpaired *t* test. *p < 0.05, **p < 0.01, ***p < 0.0001 represent significant differences.

A traditional method based on a fluorescent probe named DCFDA was used to compare with T1 relaxation. The measurement is based on 2′,7′-dichlorofluorescein (DCF), which forms a fluorescent compound (2′,7′-dichlorodihydrofluorescein diacetate (H_2_DCFDA)) when reacting with ROS (Figure 5a). LPS, as a potent macrophages activator,^44^ can stimulate ROS generation. Besides, both *S. aureus* and *S. aureus*–FND conjugates significantly (*P* < 0.05) increased ROS production compared to the control group, which indicates that *S. aureus* can activate macrophages. It has been proven that this activation is induced by lipoproteins, lipoteichoic acid, and other staphylococcal molecules.^45^ The concentration of FNDs used in this experiment alone did not increase ROS production compared with the untreated macrophages (Figure S6).

On top of that, *S. aureus* is known to produce numerous proteins (superoxide dismutase, denitrosylase, or catalase) to catabolize ROS, specifically, NO*, H_2_O_2_, and O_2_^–^*.^4^ When LPS stimulated macrophages were treated with *S. aureus*, the cellular ROS was also evaluated to test how *S. aureus* modulate the higher oxidative stress. Surprisingly, a higher fluorescent intensity was recorded in this group, compared with the LPS stimulation or the *S. aureus* infection groups. The DCFDA assay fails to differentiate the ROS molecule types and measures the accumulation of all intracellular ROS.

### 2.4 Survival of Intracellular *S. aureus*

The gold standard for bacterial survival is the capacity to form colonies. ROS have a microbicidal activity. However, different bacterial strains have different resistances to ROS.^46,47^ Intracellular *S. aureus* were extracted and cultured on TSB agar plates for CFU counting, to investigate whether LPS stimulated macrophages have an influence on intracellular *S. aureus*. Figure 5b shows that LPS stimulated macrophages can slightly inhibit the growth of bacteria from 1 h post infection compared with nonstimulated macrophages. In combination with the DCFDA results, the extra ROS produced by LPS stimulation may be a reason for this inhibition, which is also supported by the work from Rowe et al.^48^ At MOI 20, the intracellular bacteria persist even in a higher ROS environment as shown in Figure 5b. Bacteria kept a constant survival rate with or without LPS stimulated macrophages 1 h postinfection. Bacteria have evolved mechanisms to withstand ROS, and multiple proteins of the stress response would ensure bacterial survival in phagosomes.^6,7^ Besides, the influence of phagosomal acidic and nutrients insufficient environment did not induce any bacteria death until 48 h (Figure S2).

### 2.5 Free Radical Detection by T1 Relaxation

**Figure 6:**
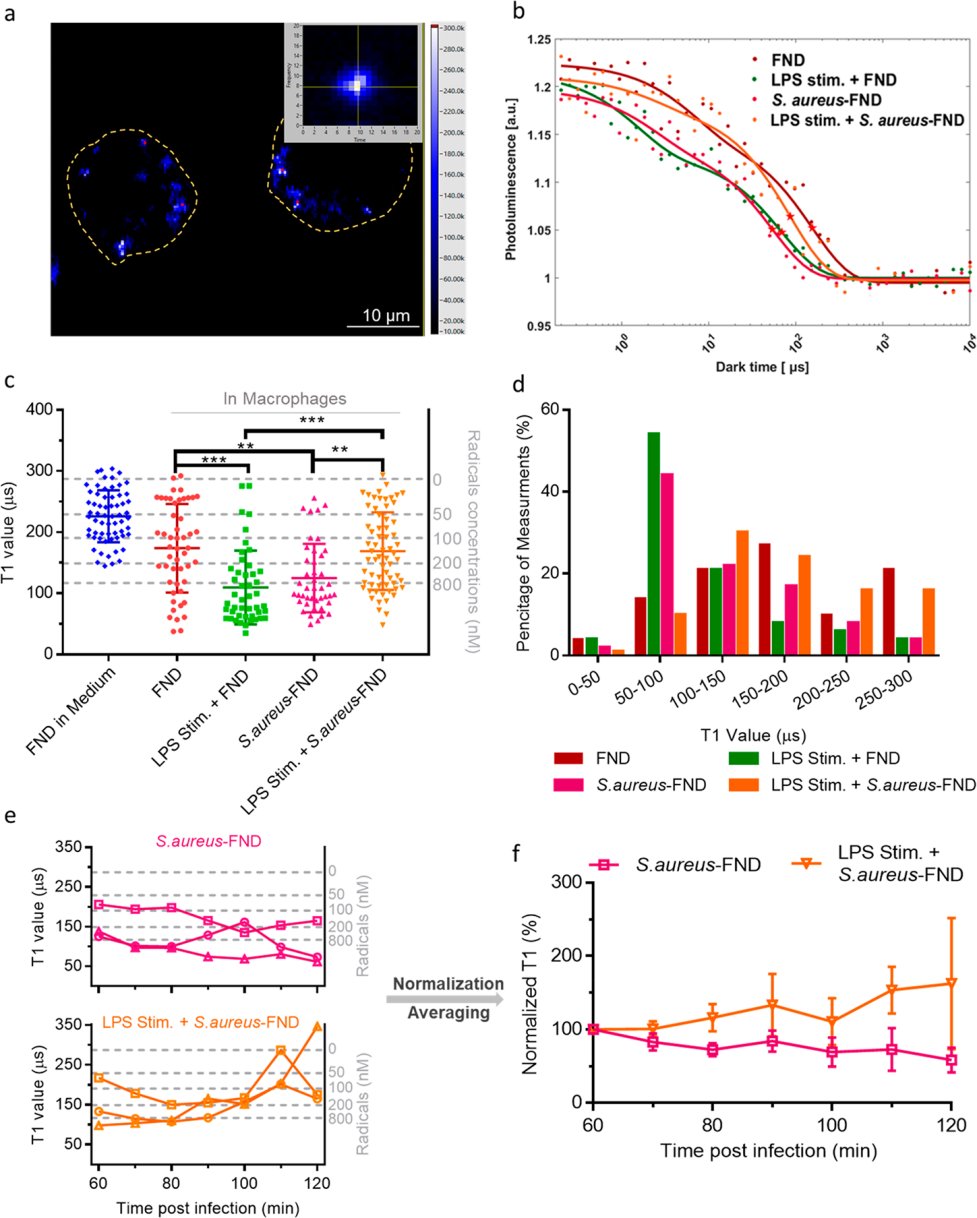
Free radical detection by T1 relaxation in macrophages in combination with LPS and/or *S. aureus*–FND. (a) Representative fluorescence image of *S. aureus*–FND in macrophages. The dashed line is the cell border. Inset: T1 relaxation measurement and refocus on the chosen FND particle during the measurement. The inset window size is 1.5 μm × 1.5 μm. (b) Typical T1 relaxation curves of FND or *S. aureus*–FND in macrophages with and without LPS stimulation. The solid lines are the biexponential fit to the measured data. (c) T1 relaxation time of each measurement was extracted from the recorded data by biexponential fitting. In addition, FNDs in radical free medium were used as the blank group. The right Y axis represents the approximated radical concentration determined from an estimation with OH* in solution from previous work.^56^ The T1 values vs known concentration of radicals and is presented in Figure S8. Nonstimulated macrophages treated with FND were set as a control group. For LPS stimulated cells, 2 μg/mL LPS was incubated with macrophages for 24 h to stimulate more free radical generation before the addition of FND or *S. aureus*–FND conjugates. T1 measurements on FNDs in cell culture medium (cell free and radical free environment) were used as a control group. Each T1 measurement takes 5 min. The data were shown as mean ± SD and analyzed using a one-way ANOVA among groups; data between each group were analyzed by an unpaired *t* test. *p < 0.05, **p < 0.01, ***p < 0.0001 represent a significant difference. (d) T1 histogram of FND or *S. aureus*–FND conjugates in living macrophages w/wo LPS stimulation. Dotted lines represent the fitting curve of the T1 data distribution of each group. (e) T1 data obtained from *S. aureus*–FND conjugates incubated w/wo LPS stimulated macrophages at different times post infection. The right Y axis represents the approximated radical concentration. Each curve represents measurements performed on the same particle at different time. (f) Average of independent normalized T1 curves for each group. The data was presented as mean ± standard deviation.

Relaxometry is able to detect the radical response on the surfaces engulfed bacteria inside the phagosomes by using *S. aureus*–FND conjugates. As shown in the fluorescent images acquired by our homemade relaxometer (Figure 6a), the FNDs (white and red spots) are very bright in comparison to the autofluorescense of the cell. FNDs with around 3 million photon counts/s were picked for T1 relaxometry measurements. T1 relaxation times of each measurement were extract from the recorded data using biexponential fitted relaxation curves (Figure 6b). As shown in Figure 6c, cells that were incubated with *S. aureus*–FND conjugates produced shorter T1s (∼125 μs) compared with initial free radical loads (∼174 μs). This finding suggests that more free radicals (paramagnetic ROS/RNS) are generated, which is consistent with the DCFDA assays. As explained above, the macrophages of the innate immune system can recognize staphylococcal molecules, and the *S. aureus* here act as attractants for marcophages. Superoxide anions (O_2_^–^*), a major species and precursor for other ROS, have been previously shown to mediate activation.^49^ It is also clear from Figure 6c that there is a relatively large spread of T1 values in macrophages. This is consistent with two main points that are known from the literature: First, it is known that even macrophages with an identical genetical background differ greatly in their ability to combat bacteria. Reasons for this might be different local environments and differences in the cell cycle and metabolic activity.^50,51^ Also, bacteria differ in their response to stress and while some are able to produce large amounts of antioxidants to neutralize free radicals.^52^

To stimulate macrophage cells to generate more free radicals and to inhibit the growth of *S. aureus*, an identical amount of LPS (2 μg/mL) was used as in the DCFDA assay. Although there is higher ROS produced by 1 μg/mL LPS stimulated macrophages, T1 measurements are more sensitive and selective for free radical detection (Figure S6). We observed much shorter T1s (∼109 μs) when using higher LPS concentrations (2 μg/mL) to stimulate macrophages, indicating that significantly more free radicals were generated. Nitric oxide (NO*) is the main free radical (paramagnetic RNS) induced by 24 h of LPS stimulation.^53^

When there are *S. aureus* present in the 2 μg/mL LPS stimulated macrophage samples, T1 values are dramatically increased (∼170 μs) compared with LPS stimulated and *S. aureus*–FND groups, meaning that less radicals were detected, which is different from the DCFDA results. This increasing T1 could be induced by the microorganism antioxidative defense system, as it has been proven that *S. aureus* involved elaborate strategies to withstand and perturb ROS and RNS. Among them, flavohemoglobin (Hmp) of *S. aureus* acts as a denitrosylase, removing NO*,^54,55^ which is mainly elicited by LPS. Besides, staphylococcal superoxide dismutase A (SodA) and superoxide dismutase M (SodM) play roles in incapacitating superoxide radicals.^7^ Interestingly, the extra oxidative stress induced by LPS may provoke the microorganism antioxidative defense system. Relaxometry performed on *S. aureus*–FND conjugates is able to detect the dynamic radical response on the engulfed bacteria surface inside the phagosomes without being involved in the redox process, while the DCFDA assay measures the accumulation of general intracellular ROS, including paramagnetic and nonparamagnetic ROS. Hence, the DCFDA assay probably fails to detect the oxidative stress regulation by a microorganism antioxidative defense system.

In addition, high throughput is the advantage of DCFDA assays, while relaxometry can be used to investigate the radical response of a single cell or even a single bacterium. To investigate the biological varability, T1 data was collected from around 50 particles in 50 different cells or on 50 different bacteria surfaces. Figure 6d shows a T1 histogram. T1 values of macrophages that were treated with LPS or *S. aureus* are distributed in 50–100 μs (54% and 44%, respectively), while the control group and *S. aureus*–FND in LPS stimulated macrophages have T1 values mainly distributed between 100 and 200 μs (48% and 57%, respectively).

How individual bacteria regulate the oxidative stress surrounding them can be investigated more clear from the T1 measurements on the same particle at different times post infection (Figure 6e,f). In macrophages without LPS stimulation, the radical load near bacteria slightly increases, while radicals were continuously consumed in LPS stimulated macrophages from 80 min post infection due to the microorganism oxidative defense system. This result may indicate that *S. aureus* scavenged more phagocyted radicals than being produced by macrophages, to throw off the radical balance between pathogens and the host at higher oxidative stress. Besides, this balance and imbalance there might be a cycle as shown in Figure 6e,f. The fluctuations presented in cycle on all curves, while radicals scavenged by *S. aureus* are dominant when radical loads reach a certain critical value. T1 from a blank group (only FNDs in medium) is relatively constant for multiple measurements of the same diamond (Figure S9).

### 2.6 Influence of pH, Temperature, and Other Factors on T1 Relaxation

Fujisaku et al. demonstrated that the T1 relaxation of FND alters slightly upon the change in its pH environment (pH 3–11) through change the degree of ionization of the surface chemistry.^57^ Though the acidification process (down to pH 4–5) of phagosomes is involved in the antibacterial process of macrophages,^4,58^ this intraphagosomal pH progressively decreases over 15–60 min, while our diamond relaxometry measurements were performed after 1 h post infections. On the other hand, our previous work concluded that T1 was not influenced by pH significantly from pH 3 to 5.5.^33^

Similar to pH, temperature,^59,60^ molecular interaction, solvent, and solvent viscosity affect the NV coherence as well.^61^ However, all these factors are relatively stable in a cell and have been shown to be negligible in the range that is relevant in this application. Hence, their effect on changes in T1 observed during our measurements can be disregarded. Furthermore, although it has been shown that cellular ROS induced by photo illumination is often involved in the signaling pathway,^62^ the artifacts generated due to photoinduced free radical formation were safely and simply dealt with by including a control group.

### Study Limitations

The most severe limitation of our method is that it requires a (nano)diamond within a few nanometers from the region of interest. This means that, for intracellular measurements, nanodiamonds need to be ingested by cells. Additionally, measurements can only be done where particles can be directed to. So far it is also not possible to differentiate between radicals and our measurement detects the sum of all radicals. Finally, drastic changes in pH might alter the observed T1 and might need to be accounted for.

## Conclusions

Phagocytosis is the most efficient system to eliminate microbe infection; however, *S. aureus* can manipulate phagosome maturation and avoid the phagocytosis processes. Inside phagolysosomes, the oxidative environment can fail to damage *S. aureus*. A possible explanation is antioxidants from *S. aureus*, which prevent the formation of free radicals. In the past decades, several ROS-related staphylococcal proteins and enzymes were characterized to explain the microorganism’s antioxidative defense system. Yet, time-resolved and site-specific free radical/ROS detection have never been achieved.

In this work, we contribute to the understanding of the failure of phagosome killing by utilizing diamond-based quantum sensing. Here, the radical response on the surface of each pathogen was detected in the *S. aureus*–macrophages infection system. By using relaxometry, we observed that *S. aureus* infection can induce radical response of macrophages, and this kind of oxidative stress can reach the pathogen surface, while the phagosome environment of macrophages may also lead to the oxidative stress of phagocytized bacteria. However, under a high intracellular oxidative stress environment elicited by LPS, radicals are more likely scavenged or modulated by *S. aureus*. Moreover, the redox reaction-free detecting mechanism of relaxometry allows for the investigation of radical responses on the same individual pathogen at different infection times. From chronological T1 results, we concluded that macrophages incubated with *S. aureus* kept the radicals in balance. However, *S. aureus* scavenged more phagocyted radicals than were produced by macrophages when the higher oxidative stress was reached.

Going forward, efforts need to be taken to (1) investigate radical response at the earlier infectious time, when pathogens are still outside the macrophages and (2) efficiently track the movement of the FND labeled pathogen, as how pathogens move inside the host lacks investigation as well. Finally, we hope that this method will enable researchers to discover and/or redefine the fundamentals of bacterial infection and thereby contribute improve the cellular immune defense.

## 3 Methods/Experimental

### 3.1 Materials

Fluorescent nanodiamonds with a hydrodynamic diameter of 70 nm (FND70) containing >300 NV centers were purchased from Adams Nanotechnologies (Raleigh, NC). These particles are produced by HPHT synthesis followed by grinding and irradiation (with an electron beam at 3 MeV and a fluence of 5 × 10^19^ e/cm^2^) and thermal annealing (above 600 °C) by the manufacturer as described in.^38^ They are widely used for different purposes and have been extensively characterized before.^63^ We found these particles to have an ideal size for this purpose.^56^ Smaller particles are less bright, so it takes longer to obtain a good signal-to-noise ratio. In addition, they move faster and are therefore more difficult to track. However, it is probably most important that the larger particles contain more NV centers and, thus, that each measurement is already an average of all these NV centers. Hence, signals from these particles are more reproducible. The drawback of even larger particles is that NV centers in the core of these particles are too distant from the surface to sense the spin noise from radicals. A Cellular ROS Assay Kit (DCFDA/H2DCFDA, ab113851) was purchased from Abcam. We obtained lipopolysaccharide (LPS) from *E. coli* K12 (LPS, to stimuli macrophage cells) from InvivoGen. LPS Ultrapure was extracted by enzymatic hydrolysis steps and purified by a phenol-TEA-DOC extraction protocol.^64^ ELISA kits (Mouse IL-1βELISA Kit (ab197742) and Mouse IL-18 ELISA Kit (ab216165)) were purchased from Abcam (Cambridge, UK). Tryptone Soy Broth (TSB) medium was purchased from Sigma OXOID (Zwijndrecht, The Netherlands). A Live/Dead BacLight bacterial viability kit (SYTO 9/propidium iodide, L7012) was obtained from Invitrogen, Thermo Fisher Scientific (Toulouse, France). Lysoview405 (a blue-fluorescent stain for imaging lysosomes) was purchased from Biotium (Fremont, CA).

### 3.2 Cell Culture and Bacteria Preparation

J774A.1 (LGC Standards), immune cells from a mouse cell line, were cultured in complete Dulbecco’s modified eagle medium with high glucose (DMEM-HG), supplemented with 10% fetal bovine serum (FBS), 1% penicillin/streptomycin, and 1% Glutamax (Gibco, Thermo Fisher Scientific (The Netherlands)). The antibiotics penicillin/streptomycin were only used before coculturing. When cells were cocultured with bacteria, they were cultured only in glucose free Dulbecco’s modified eagle medium (DMEM), without penicillin/streptomycin. Cells were cultured in an incubator at 37 °C and 5% CO_2_. To stimulate the macrophages, 2 μg/mL LPS was added to supplemented DMEM for 20 h.

Bacterial cultures of *S. aureus* (RN4220) were prepared by suspending a colony in 10 mL of TSB medium and incubated at 37 °C for 24 h aerobically. Subsequently, 2.5 mL was transferred into 50 mL of TSB in a ratio of 1:20 (v/v) followed by 24 h of incubation at 37 °C aerobically. Next, the cultures were washed twice with 10 mL of sterile phosphate buffered saline (PBS) alternated by centrifugation at 10 °C and 4732*g* (Beckman Coulter JLA 16.250). After washing, the cultures were resuspended in PBS, followed by pulsed sonication (Sonicator rod, Vibra Cell) in an ice bath for breaking bacterial aggregates. Bacteria in the suspension were quantified with a Bürker-Türk counting chamber. Cultures of *S. aureus* (RN4220) transfected with fluorescent reporter plasmids (pMV158 green fluorescent protein)^65^ were prepared following the same protocol explained above using TSB medium supplied with 10 μg/mL tetracycline and is henceforward identified as *S. aureus*–GFP.

### 3.3 Nanodiamond–Bacteria Conjugation

To quantitatively detect free radical generation inside early phagosomes using T1 relaxation, the first step is to prepare FND–bacteria conjugates. Specifically,1 × 10^8^ bacteria of *S. aureus* were mixed with 5 μg of FNDs in 100 μL of demineralized water (demi water) and placed at 37 °C in a shaking incubator at 150 rpm for 2 h. A total amount of 1 mL of demi water was added to this mixture, followed by centrifuging at 10 °C and 7000*g* for 10 min. The pellet was then resuspended using 100 μL of demi water. Then, a 10 times dilution of this suspension was placed into a Petri dish for imaging. The samples were imaged using a confocal microscope (Zeiss LSM780 NLO (from Zeiss, Germany)). To obtain the conjugation efficiency with FNDs by confocal microscopy, *S. aureus*–GFP was used. The images were analyzed using FIJI^66^ (https://fiji.sc/) to determine the conjugation efficiency between *S. aureus* and FNDs. To obtain a more detailed insight of the conjugate morphology, scanning electron microscopy (SEM) was used. To prepare samples for SEM, 10 μL of *S. aureus*–FNDs conjugates was dropped onto a silicon wafer and was left for 10 min. Then, excess water was drained and the sample was dried. The sample was then imaged by a S/TEM instrument (FEI Helios G4 CX, Hillsboro, OR) at 3 kV with both a circular backscatter detector (CBS) and through the lens detector (TLD).

Dynamic light scattering (DLS) was used to investigate the size distribution of FNDs in different bacteria culture media (demi water, 30% TSB, 100% TSB); the measurements were performed at 25 °C using a Zetasizer Nano ZS ZEN3500 (Malvern Instruments, The Netherlands) equipped with a 633 nm He–Ne laser using backscattering detection.

### 3.4 Bacterial Viability Test

To determine if bacteria can survive at the conjugation condition mentioned earlier, the bacteria viability was accessed by a live/dead staining. *S. aureus* (1 × 10^9^ bacteria/mL, 1 mL) were kept in TSB and demi water in a 12-well plate, respectively. After 2 h of incubation at 37 °C and in a shaking incubator at 150 rpm, the nonadhered bacteria were removed and washed three times with sterile PBS. After that, samples were stained with 300 μL of a BacLight Live/Dead viability kit containing SYTO 9 (5 μM) (yielding green fluorescence in live organisms) and propidium iodide (30 μM) (yielding red fluorescence in membrane-damaged, dead organisms). Subsequently, we incubated for 15 min in the dark. After staining, the samples were imaged using a fluorescence microscope (Leica DM4000B (Leica, Germany)). Specifically, live bacteria were imaged using a GFP filter (Ex/Em: 457–487/502–538 nm) and dead bacteria were imaged using a N21 red filter (Ex/Em: 515–560/625–780 nm), respectively. Images were quantitatively analyzed using the FIJI software. Three different images were recorded from three different locations for every well in each experiment. Bacteria cultured in TSB were used as control groups; the experiment was repeated three independent times.

To test the viability of *S. aureus* inside the acidic environment (pH = 5.0) in endosomes of macrophages, 1 × 10^8^ bacteria/mL *S. aureus* were kept in 1 mL of TSB, glucose free DMEM, and pH 5.0 glucose free DMEM in a 12-well plate, respectively. After 2, 24, and 48 h incubation, samples were stained with a Live/Dead kit and imaged using a fluorescence microscope (Leica DM4000B). Three different images were recorded from three different locations of every well for each experiment. Bacteria cultured in TSB were used as control groups to set up the fluorescent microscope. The number of live and dead bacteria were counted to obtain the bacterial viability under acidic conditions (pH = 5.0). The experiment was repeated three independent times.

### 3.5 Subcellular Location of *S. aureus*–GFP–FNDs Conjugates

Before a free radical measurement, one must know where exactly within a macrophage cell *S. aureus*–FNDs conjugates are located. To this end, 2 × 10^4^ macrophages in DMEM glucose free medium were incubated with the *S. aureus*–GFP–FNDs conjugates (multiplicity of infection (MOI) = 20)) for 30 min. After incubation, the cell medium was removed, and cells were washed with PBS. Then, we stained the cells with lysosome tracker (Lysoview405, in blue), which stains phagosomes or lysosomes. Samples were imaged with a Zeiss confocal microscope; FNDs were detected at Ex/Em = 561/659 nm, lysoview405 at Ex/Em = 358/461 nm, and GFP at Ex/Em = 495/510 nm. Z scans were used to confirm that *S. aureus*–GFP–FNDs conjugates are located inside cells. These images were processed using the FIJI software.

### 3.6 Growth of Intracellular *S. aureus*

*S. aureus* were used to infect macrophages with or without LPS pretreatment (10^5^ macrophages/well) in 24-well plates (Cell Culture-Treated, Flat-Bottom Microplate, Greiner Bio-One (Amsterdam, The Netherlands)) at a MOI of 20. The cells were washed once with PBS at 30 min post infection. Fresh medium containing 30 μg/mL gentamicin (Sigma-Aldrich (Zwijndrecht, The Netherlands)) was added to kill the extracellular bacteria in the wells. At the indicated times (0.5, 1, 2, and 4 h post infections), 200 μL of Triton X-100 (0.1%) in aqueous solution was added to each well for 5 min at 37 °C to selectively lyse the macrophages and release the bacteria. PBS (800 μL) was added to each well, and the lysates were suspended by pipetting. The colony forming units (CFUs) were determined by serially diluting the samples in PBS and plated on a TSB agar plate. The CFUs of each group at the indicated times were enumerated after 24 h incubation at 37 °C. The averages and SD of three biological replicates are shown (*n* = 3).

### 3.7 DCFDA Assay

Two ×10^4^ macrophages per well were seeded in a 96-well microplate (Cell Culture-Treated, Flat-Bottom Microplate, Greiner Bio-One) and incubated overnight to allow for attaching to the bottom. After incubation, cells with or without (w/wo) LPS stimulation were washed with PBS and stained with 10 μg/mL DCFDA for 45 min. After staining, cells were washed, and the medium was replaced with PBS. Then, the cells were incubated with *S. aureus* (MOI = 20), *S. aureus*–FNDs conjugates (MOI = 20), and FNDs (identical concentration as *S. aureus*–FNDs conjugates) for 1 h. After incubation, the fluorescence intensity was measured by a plate reader (Bio Tek, Santa Clara, CA) at Ex/Em = 485/535 nm in end point mode. Cells without staining with DCFDA were recorded for background subtraction.

### 3.8 T1 Measurement

To perform diamond relaxometry, we utilized a homemade magnetometry setup. The setup is in principle a confocal microscope with a few adaptations.^36,67^ The laser we used is a 532 nm laser at 50 μW at the location of the sample (measured in continuous illumination). This sequence consisted of 5 μs long laser pulses separated by variable dark times τ from 0.2 to 10 000 μs. We used a ×100 magnification oil objective (Olympus, UPLSAPO 100XO, NA 1.40) and an acousto-optical modulator (Gooch & Housego, model 3350-199) to conduct the pulsing sequence.

Using a bright field camera (Thorlabs), we selected FND particles well inside a cell with a brightness around 3 million photon counts/s. These nanodiamonds are very well-characterized in the literature.^68^ We then confirmed that the fluorescence is stable (bleaching structures are background fluorescence rather than FNDs) and performed a Z-stack to confirm that the particle is indeed within a macrophage. T1 measurements were performed with glucose free DMEM medium to prevent *S. aureus* to over grow inside macrophages and thus avoid biofilm formation. In a T1 or relaxometry measurement, we bring a NV center in the bright *m*_S_ = 0 state of the ground state and determine how long it takes to return to the darker equilibrium. The pulsing sequence was repeated 5000 times for each measurement to obtain a sufficient signal-to-noise ratio. The purpose of each pulse was to measure if the NV centers are already in the equilibrium and pump them into the ground state again. If there are radicals (essentially unpaired electron spins) in the surrounding, this process will occur faster. This optically detected T1 time is equivalent to a T1 signal in conventional MRI. However, NV centers can only sense their immediate surrounding the signal originates from nanoscale voxels around the particles (estimated from scanning magnetometry experiments to be below 100 nm from the NV centers^69^). The duration of all the measurements and repetitions combined took around 5 min.

For T1 measurements of free radicals generated inside phagolysosomes of macrophages, 2 × 10^4^ cells were seeded in a glass bottom Petri dish. To avoid biofilm formation, glucose free DMEM medium was used for T1 measurements. Cells incubated with FNDs for 1 h were used to measure the initial number of free radicals inside a macrophage cell. Cells were pretreated with LPS for 24 h; then, cells, which were incubated with FNDs for 1 h, were used to detect the number of free radicals in a LPS stimulated macrophage cell. Macrophages incubated with for 1 h were used to measure the free radicals generated by *S. aureus* infected macrophage cell. In addition, in order to know how *S. aureus* regulate higher radical loads, T1 experiments were performed on *S. aureus*–FNDs conjugates in LPS stimulated macrophages. T1 measurements on the same particles from the same bacteria in the same macrophages were also performed by the same procedure to investigate how individual bacteria experience a radical response in their surroundings.

## Supporting Information Available

Figures of size distribution of FNDs measured by DLS, data on bacterial viability, high resolution SEM images, and DCFDA data for comparison with T1 and to confirm biocompatibility of FNDs (PDF)
