# Supplementary material for: Diamond Relaxometry as a Tool to Investigate the Free Radical Dialogue between Macrophages and Bacteria
Source: ACS Nano. 2023 Jan 11;17(2):1100–11. doi: 10.1021/acsnano.2c08190 (PMC9878971; doi:10.1021/acsnano.2c08190)
Supplement: Supplementary file 1 — nn2c08190_si_001.pdf [file nn2c08190_si_001.pdf]

Supporting information

Diamond Relaxometry as a tool to investigate the free radical dialogue between macrophages and bacteria

Kaiqi Wu[1#], Linyan Nie[1#], Anggrek C. Nusantara[1], Willem Woudstra[1], Thea Vedelaar[1], Alina Sigaeva[1], Romana Schirhagl[1*]

1 University of Groningen, University Medical Center Groningen, Department of Biomedical Engineering, Antonius Deusinglaan 1, 9713 AV, Groningen, The Netherlands

*Corresponding author. Email: romana.schirhagl@gmail.com.

# These authors contribute equally.

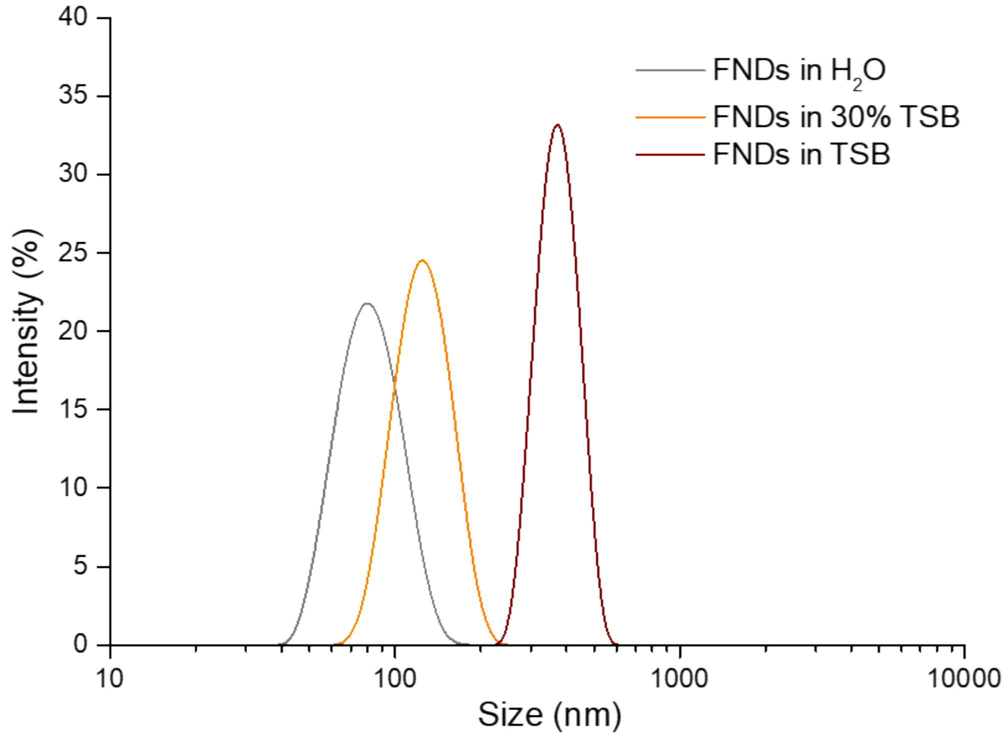

Figure S1. Size distribution of FNDs in ultrapure water, 30% TSB, and TSB measured by DLS.

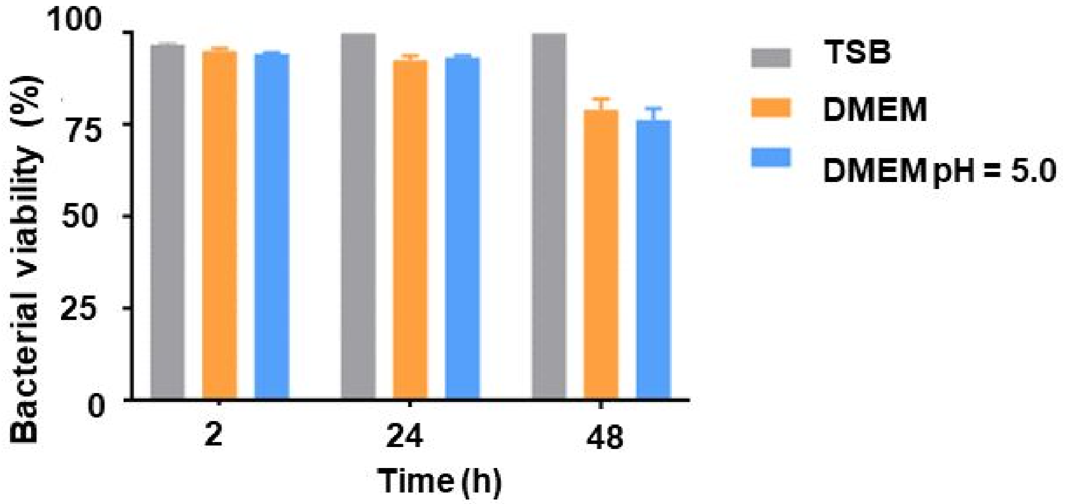

Figure S2. *S. aureus* viability tested in TSB (used as a control), DMEM cell culture medium (pH = 7.0), and DMEM (pH = 5.0 to mimic the acidic environment in phagosomes) cell culture medium at different time points. DMEM glucose free medium was used in this experiment to keep identical condition as in the following T1 measurements.

An acidic environment was reported as a key factor for *S. aureus* surviving from macrophages as described above, here we tested *S. aureus* viability in different culture conditions. To mimic the acidic environment inside phagolysosomes, DMEM glucose free medium (pH = 5.0) was chosen to culture bacteria. TSB (pH = 7.0) and DMEM glucose free medium (pH = 7.0) were used to culture bacteria. We kept the same conditions for free radical detection by T1 relaxation. **Figure S2** shows that *S. aureus* can survive in acidic DMEM medium even after 48 h, which confirms that acidic environment alone did not kill *S. aureus*.

a

b

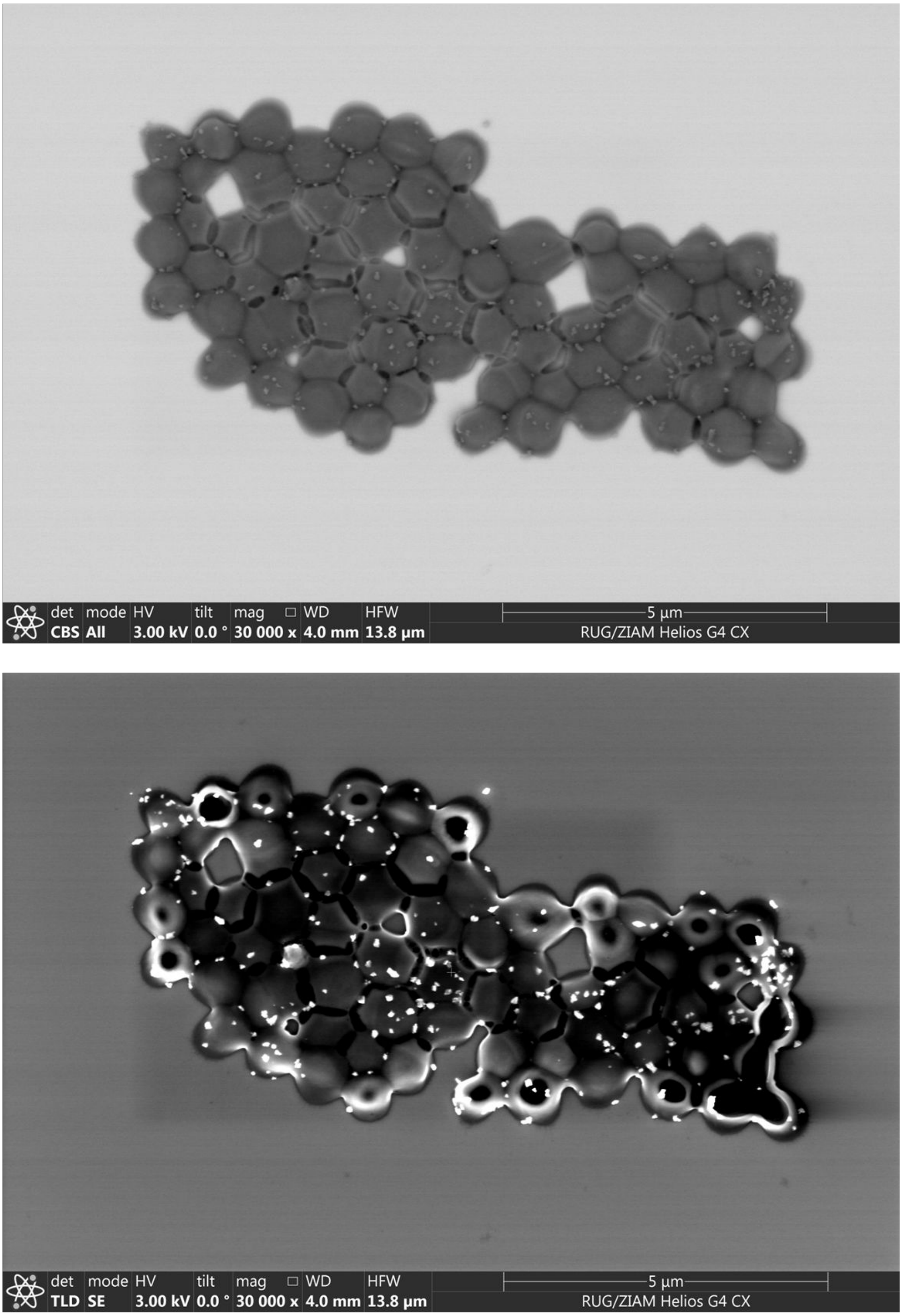

Figure S3. SEM images of *S. aureus*-FNDs conjugates by CBS detector (a) and TLD detector (b) with 30 000x magnification at 3 kV.

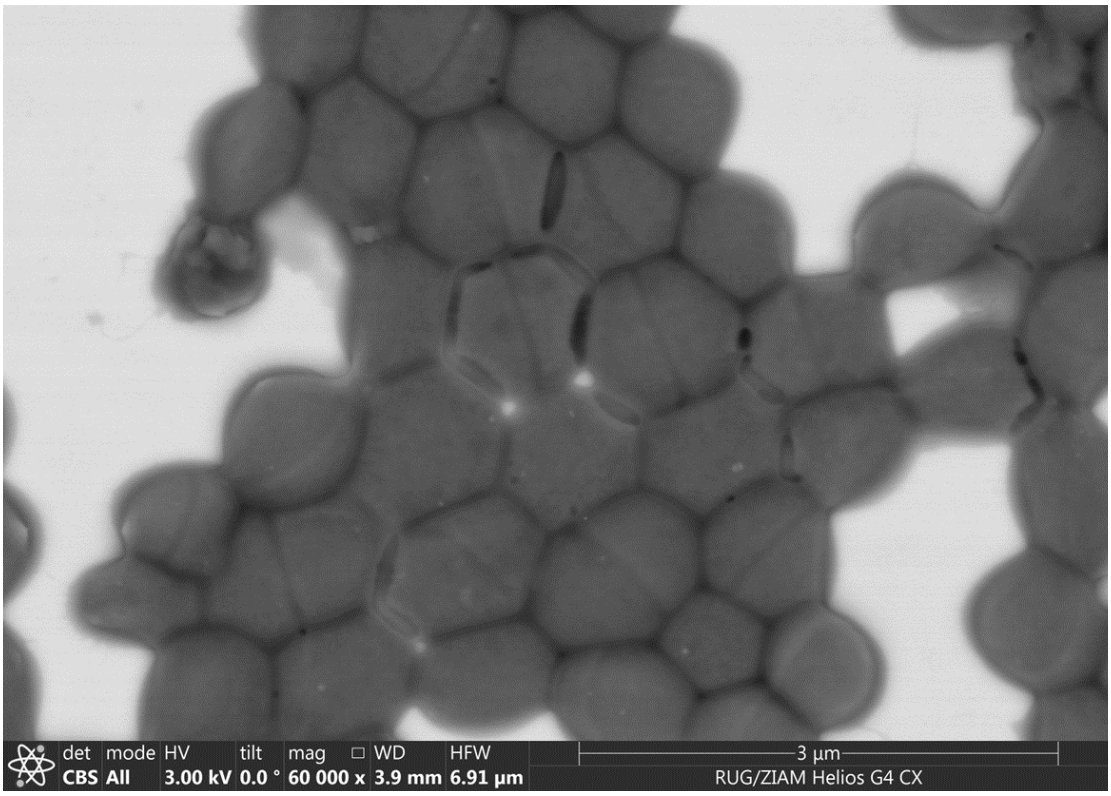

Figure S4. SEM images of *S. aureus* by CBS detector with 60 000x magnification at 3 kV.

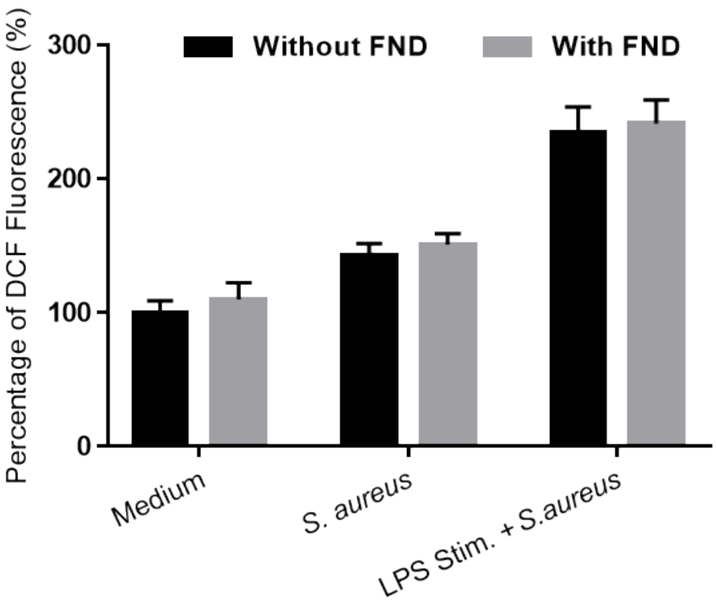

Figure S5. DCFDA assay of w/wo stimulated macrophages when treated by bare FND in cell culture medium, *S. aureus*-FND.

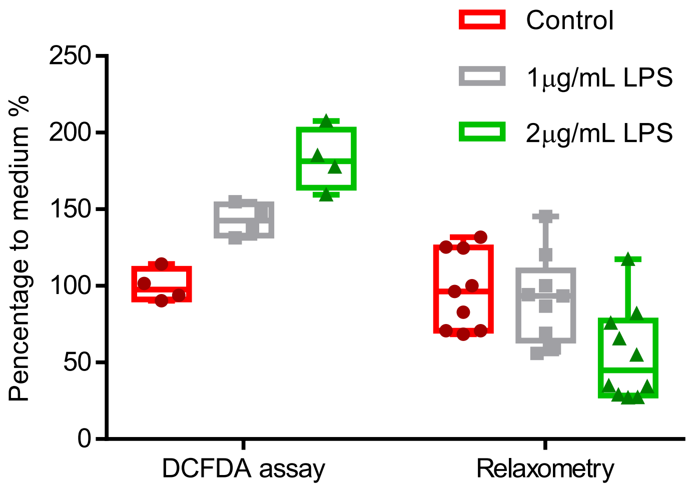

Figure S6. DCFDA assay and diamond relaxometry when macrophages are treated with cell culture medium (set as control group), 1 μg/mL LPS, and 2 μg/mL LPS.

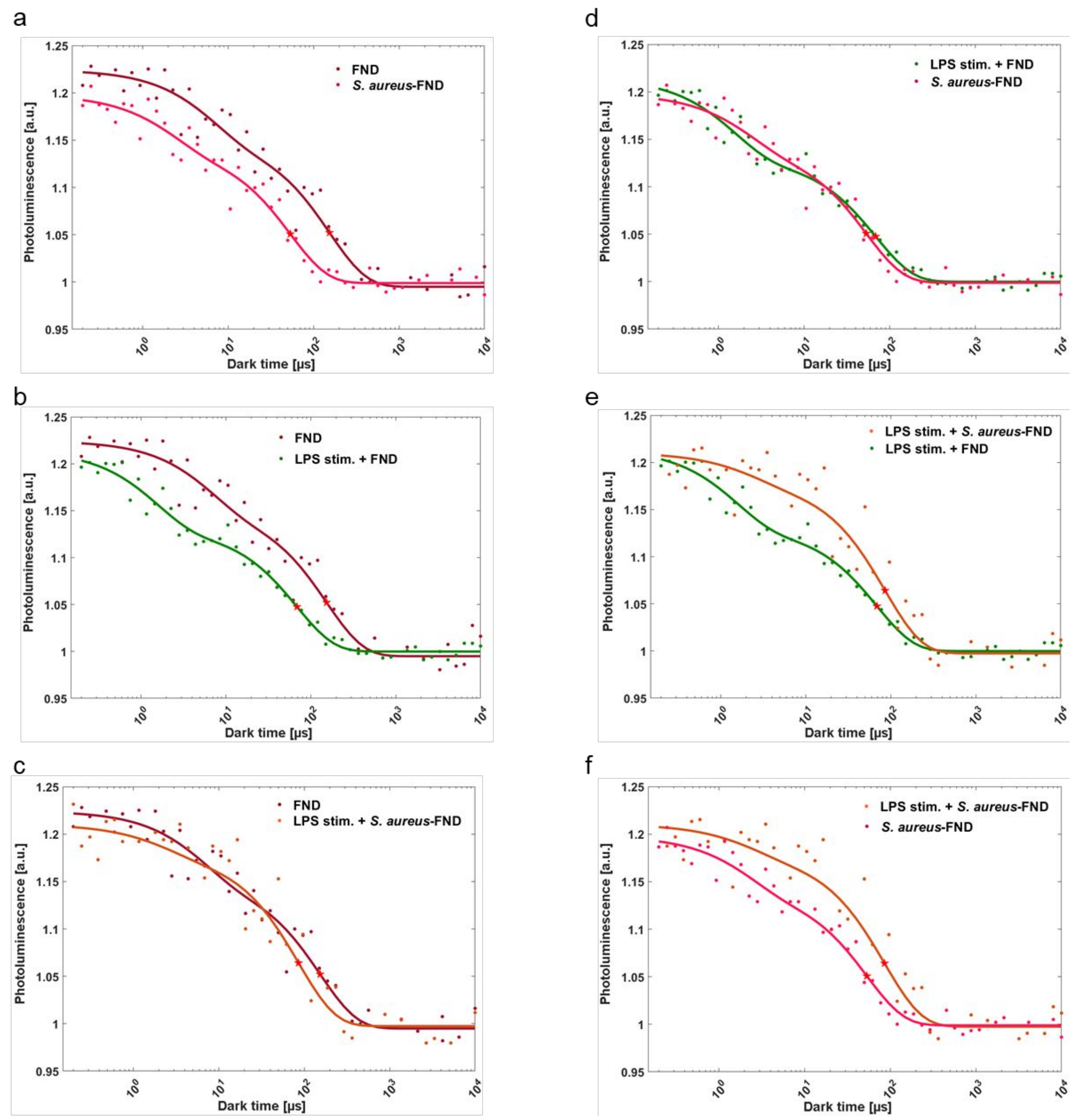

Figure S7. Comparisons of typical T1 relaxation curves of each group in macrophages. The solid lines are the biexponential fit to the measured data. (a) FND *vs. S. aureus*-FND in unstimulated macrophages; (b) FND in unstimulated macrophages *vs.* FND in LPS stimulated macrophages; (c) FND in unstimulated macrophages *vs. S. aureus*-FND in LPS stimulated macrophages; (d) FND in LPS stimulated macrophages *vs. S. aureus*-FND in unstimulated macrophages; (e) FND *vs. S. aureus*-FND in LPS stimulated macrophages; (f) *S. aureus*-FND in unstimulated macrophages *vs.* in LPS stimulated macrophages.

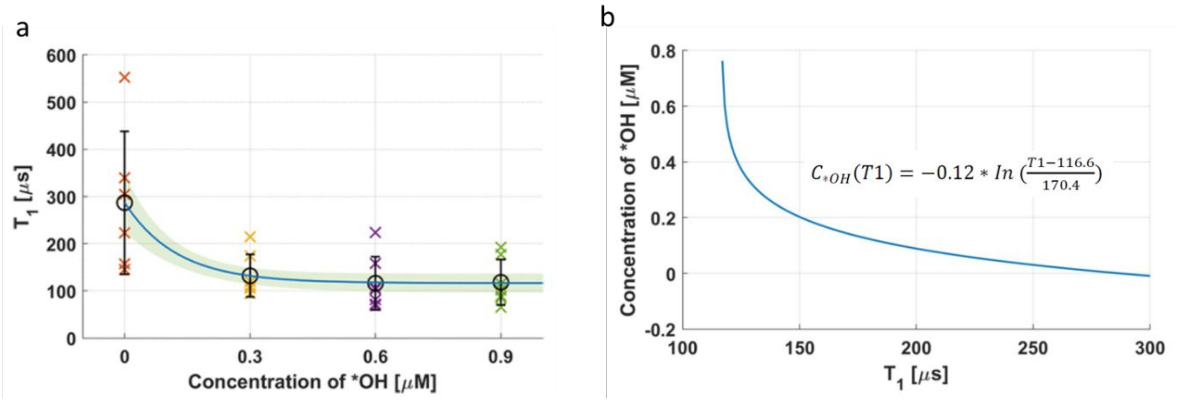

Figure S8. Estimating concentrations from T1 measurements. (a) The T1 value of an ensemble of NV centers decreases exponentially (blue line) as the concentration of *OH increases which *OH produced by photolysis of hydrogen peroxide and confirmed by 2-Hydroxy Terepthalic Acid (HTA). (b) Inverse of the curve presented in Figure a . (This calibration curve was reproduced from our previous work by F. Perona et. al (Nusantara, A.C., Martínez, F.P., Chipaux, M., Padamati, S.K. and Schirhagl, R., Nanodiamond relaxometry-based detection of free-radical species when produced in chemical reactions in biologically relevant conditions. ACS sensors 5 (12), 3862-3869))

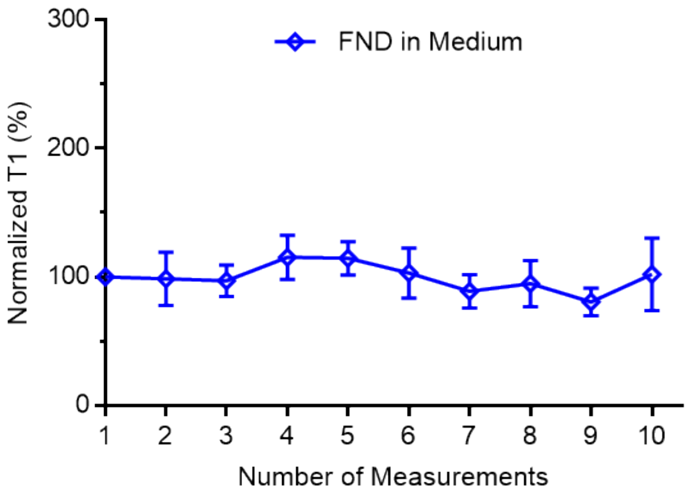

Figure S9. Relative fluctuations of T1 on the same FND particles in radical free medium. T1 values for different number of measurements were normalized to the initial T1 values of each FND particles. The data was present as mean ± S.D (n=3).
